# Oral rehabilitation with tilted dental implants: A metaanalysis

**DOI:** 10.4317/medoral.17674

**Published:** 2012-02-09

**Authors:** Javier Ata-Ali, David Peñarrocha-Oltra, Eugenia Candel-Marti, Maria Peñarrocha-Diago

**Affiliations:** 1 DDS. Primary Public Health Service Dentist. Valencian Health Service. Master in Oral Surgery and Medicine. Master in Oral Surgery and Implantology. Valencia University Medical and Dental School; 2 DDS. Resident of the Master in Oral Surgery and Implantology. Valencia University Medical and Dental School; 3 Associate Professor of Oral Surgery. Master in Oral Surgery and Implantology. Valencia University Medical and Dental School. Valencia (Spain)

## Abstract

Objective: To compare the course of patients treated with tilted implants versus those treated conventionally with axial implants, analyzing the success rate and marginal bone loss. Material and Methods: A PubMed search was made using the key words “tilted implants”, “angled implants”, “angulated implants”, “inclined implants” and “maxillary atrophy.” A review was made of the articles published between 1999-2010. The inclusion criteria were the use of tilted implants, clinical series involving at least 10 patients, and a minimum follow-up of 12 months after prosthetic loading. The exclusion criteria were isolated clinical cases, studies with missing data, and publications in languages other than English or Spanish. The metaanalysis finally included 13 articles: 7 retrospective studies and 6 prospective studies. Results: On analyzing the success rate in the retrospective studies, two reported a higher success rate with tilted implants; one a higher success rate with axial implants; and two reported similar success rates with both implants. On analyzing the success rate in the prospective studies, two reported a higher success rate with tilted implants; two a higher success rate with axial implants; and two reported similar success rates with both implants. On examining marginal bone loss, three studies reported greater bone loss with axial implants and one with tilted implants. Conclusions: There was no evidence of differences in success rate between tilted and axial implants in either the prospective or retrospective studies subjected to review. The marginal bone loss observed with the tilted and axial implants likewise proved very similar. It thus can be deduced that tilted implants exhibit the same evolutive behavior as axial implants.

** Key words:**Axial implants, tilted implants, maxillary atrophy, tilted implants.

## Introduction

The term tilted implants refers to implants placed at an angle of normally 30 degrees or more with respect to axially or vertically positioned implants (1). According to many authors, the use of tilted implants in the posterior maxillary sector offers advantages over axial implants (2-7).

The placement of tilted implants offers both surgical and prosthodontic benefits. In effect, the combination of tilted and axial implants allows the use of longer implants, thereby increasing the osseointegration surface; improves primary stability by anchoring in more than one cortical layer; avoids cantilever extremities by placing the implants more distal and with better load distribution over the dental arch; and avoids the use of bone grafts and sinus lift procedures - with the resulting reduction in morbidity (1,8).

The present metaanalysis compares the course of patients treated with tilted implants versus those treated conventionally with axial implants, analyzing the success rate and marginal bone loss.

## Material and Methods

A PubMed search was made using the key words “tilted implants”, “angled implants”, “angulated implants”, “inclined implants” and “maxillary atrophy.” A review was made of the articles published between 1999-2010. A manual search was also made, using those references to review articles considered to be important.

In selecting the publications we reviewed the titles and abstracts to identify the relevant studies, which were then retrieved in full format and assessed for the following inclusion criteria: the use of tilted implants, clinical series involving at least 10 patients, and a minimum follow-up of 12 months after prosthetic loading. The exclusion criteria in turn were: isolated clinical cases, studies with missing data, and publications in languages other than English or Spanish.

The initial search yielded 118 publications, and the first analysis based on the titles and abstracts reduced this number to 22 articles. In-depth evaluation of the full text of these papers in turn yielded 13 publications (7 retrospective studies and 6 prospective studies), which were finally included in the metaanalysis.

The differences between tilted and axial implants in terms of success rate and marginal bone loss were statistically analyzed via three metaanalyses summarized in ( Table 1). In relation to success rate, two study subgroups were established according to the design involved (prospective or retrospective), and an independent metaanalysis was carried out in each of them. Metaanalysis I examined the success rate in four retrospective studies (2,3,5,9), with the exclusion of three studies (13-15) that failed to compile data on axial implants. Metaanalysis II in turn analyzed the success rate in 6 prospective studies (4,7,8,10-12), while metaanalysis III analyzed marginal bone loss in 5 studies, though yielding QH = 11.705 for the heterogeneity test, with p = 0.019 (i.e., these were very heterogeneous studies from the statistical perspective). The source of such heterogeneity was identified as the publication by Calandriello et al. (4), which involved the smallest sample size, and reported exceptionally low marginal bone loss in tilted implants. This article was therefore excluded, leaving a final total of four studies (7,10-12).

**Table 1:**
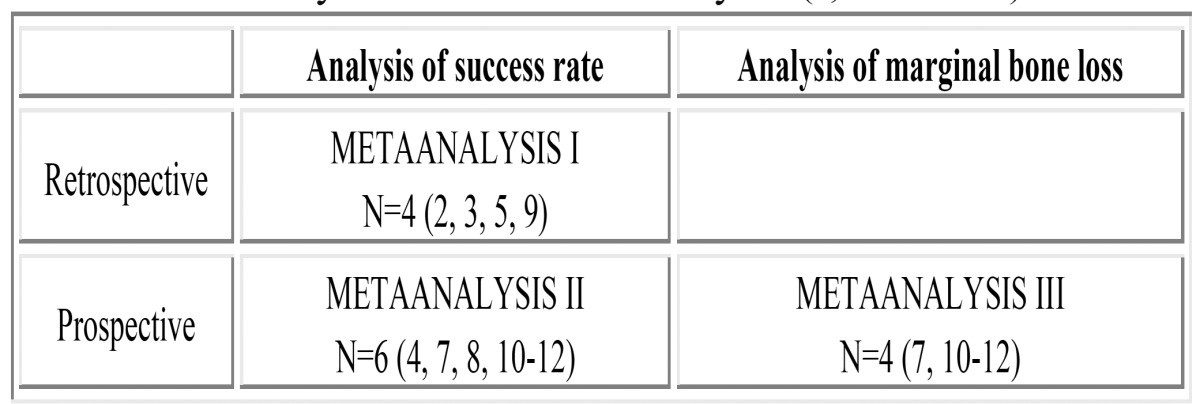
Summary of the three metaanalyses (I, II and III).

In the study of implant success rate (metaanalyses I and II) the odds ratio (OR) was used as measure of effect, since it is the most stable statistic in situations of sample size variations. The corresponding 95% confidence interval (95%CI) was reported in all cases, along with the standard error of the Naperian logarithm of the OR. In the study of marginal bone loss (metaanalysis III) we used the difference of means as the measure of effect, together with the corresponding 95%CI and the standard error of the difference of means. In both cases the random effect model was used. As calculation method we employed inverse variance of DerSimonian and Laird. The heterogeneity test was based on the QH statistic, with chi-squared distribution and k-1 degrees of freedom (k = number of studies). The global effect magnitude test was based on the distribution of the QA logic association statistic. The level of significance considered in the association and heterogeneity contrasts of the metaanalyses was 5%.

## Results

Success rate

**Figure 1:**
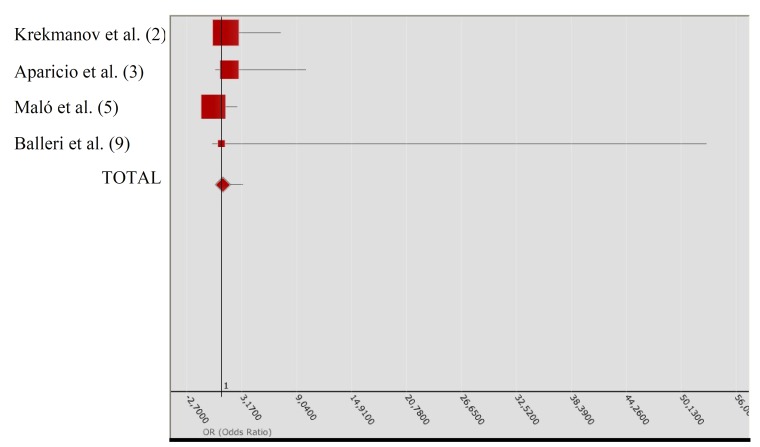
The Forest plot compares the different studies included in the metaanalysis I (measures of OR and 95%CI).

The results of metaanalysis I are presented in ( Table 2). Based on the corresponding ORs, the included studies show some contradiction. In effect, the studies of Krekmanov et al. (2) and Aparicio et al. (3) point to a greater success rate with tilted implants, while in contrast the study of Maló et al. (5) reports superior results with axial implants. In the sample of Balleri et al. (9), the descriptive results were identical in both groups, and OR = 1. The heterogeneity test showed homogeneity among the studies. The total OR was 1.162, with 95%CI. The QA association statistic was 0.079, with a p-value of 0.778, allowing us to assume the existence of homogeneity in the success rate between tilted and axial implants. The Forest plot (Fig. 1) compares the different studies (measures of OR and 95%CI).

**Table 2:**
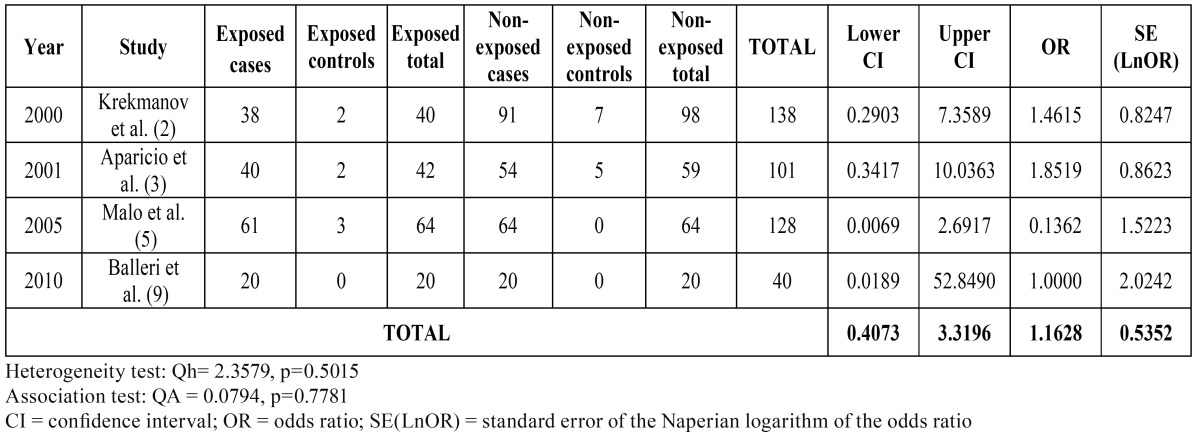
Metaanalysis I: Success rates in retrospective studies.

**Figure 2:**
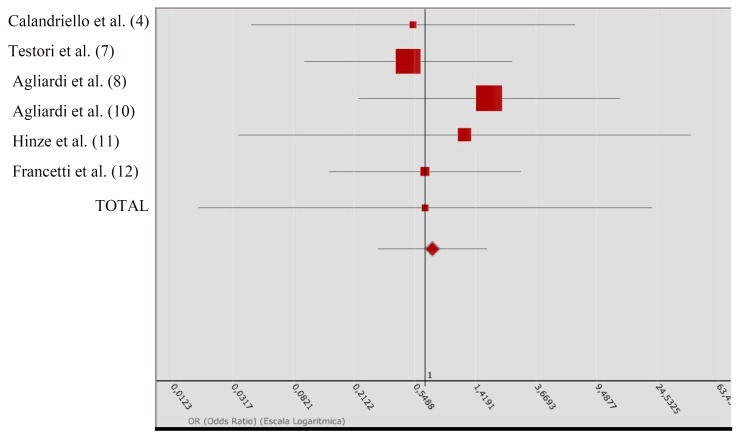
The Forest plot compares the different studies included in the metaanalysis II (measures of OR and 95%CI).

In metaanalysis II ( Table 3), the individualized ORs again indicated slightly conflicting tendencies. The studies of Agliardi et al. (8,10) indicated a greater success rate with tilted implants. In contrast, Calandriello et al. (4) and Testori et al. (7) reported superior results with axial implants. In the samples analyzed by Hinze et al. (11) and Francetti et al. (12), the descriptive results were identical, with no failures in balanced groups of tilted and axial implants (hence OR = 1). The recorded QH = 1.089 for the heterogeneity test, with p = 0.9550, indicates that the studies were quite homogeneous from the statistical perspective. The total OR was 1.137, with 95%CI. The QA association statistic was 0.071, with a p-value of 0.789, allowing us to assume the existence of homogeneity in the success rate between tilted and axial implants. The Forest plot (Fig. 2) compares the different studies (measures of OR and 95%CI).

**Table 3:**
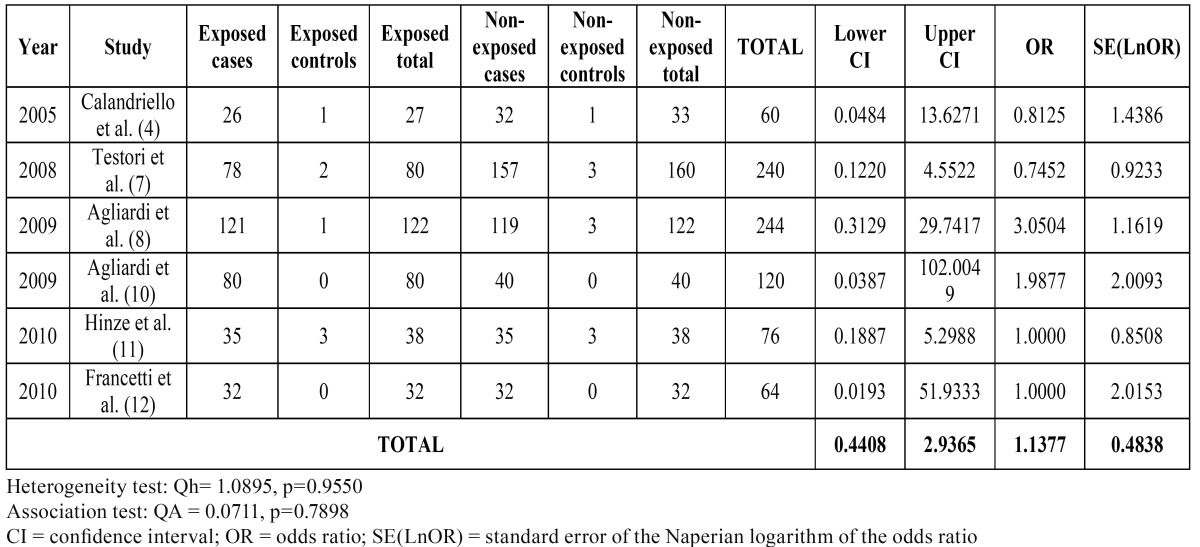
Metaanalysis II: Success rates in prospective studies.

Marginal bone loss

**Figure 3:**
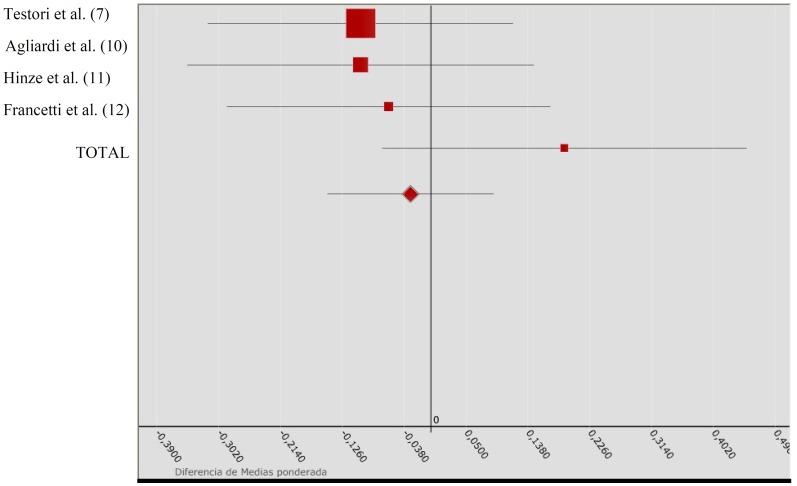
Presents the Forest plot comparing the studies included in this metaanalysis III.

Metaanalysis III relating to marginal bone loss in prospective studies yielded QH = 7.601 for the heterogeneity test, with p = 0.055. The results are shown in ( Table 4), while (Fig. 3) presents the Forest plot comparing the studies included in this metaanalysis. The studies can be taken to be homogeneous, though the work of Francetti et al. (12) proved relatively heterogeneous with respect to the rest. The differences in weighted means obtained for each individual study proved negative for the first three publications (indicating increased losses with axial implants) and positive only for the study by Francetti et al. (12). The global difference in weighted means was -0.029, with a 95%CI that clearly contained the value zero, i.e., no effect was recorded. In turn, QA = 2.457 with p = 0.117, which leads us to the same conclusion: tilted implants show the same behavior as axial implants in terms of marginal bone loss.

**Table 4:**
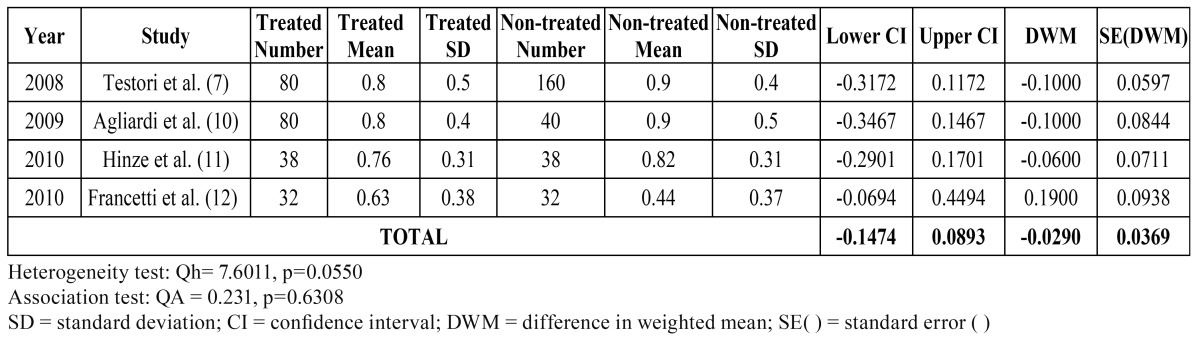
Metaanalysis III: Marginal bone loss in prospective studies.

## Discussion

In the present study it has been assumed that the analytical units in the different studies are the implants, not the randomization units (i.e., the patients). The results therefore would also indicate widening of the confidence intervals, and thus reinforcement of the conclusion regarding the homogeneity between tilted and axial implants.

Prosthetic rehabilitation of the edentulous maxilla includes the placement of tilted implants as a relatively recent option. The advantages of tilted implants are: (a) the use of longer implants, thereby increasing the contact (osseointegration) surface; (b) improved primary stability by anchoring in more than one cortical layer; (c) the avoidance of cantilever extremities by placing the implants more distal and with better load distribution over the dental arch; and (d) avoidance of the use of bone grafts and sinus lift procedures - with the resulting reduction in morbidity (1,8).

It has been considered that loaded tilted implants can fail due to the presence of unfavorable forces applied to the bone surrounding the implants. However, this theory was rejected by Celleti et al. (16) whom used these implants splinted so as to adequately distribute prosthetic loading.

In the year 2009 Agliardi et al. (10) published the largest series to date, with 61 rehabilitated maxillas in which four implants were placed: two more anterior in an axial position and two more posterior in a tilted position parallel to the anterior wall of the maxillary sinus. The success rate was 100% for both the axial and the angled implants, after a mean follow-up of 27.2 months. Peñarrocha et al. (15) in turn rehabilitated 10 patients with overdentures on four tilted implants. Only one implant failed, after 12 months of follow-up, the corresponding success rate being 97.7%.

Maló et al. (5) published a study of 32 patients with the placement of 128 dental implants (64 angled and 64 axial), the reported success rate being 95.3% and 100%, respectively. The marginal bone loss was 0.9 mm on average, with no differences between the tilted implants and the axial implants. Rosen and Gynther (14), in a study involving follow-up for as long as 12 years, with the placement of 103 tilted implants, recorded a success rate of 97%. Their mean marginal bone loss was 1.2 mm. These authors concluded that angled implants placed in the extremities of atrophic maxillas constitute a viable and evidence-based treatment option, and may be viewed as an alternative to bone grafting.

Based on the findings of our metaanalysis, there is no evidence of differences in success rate between tilted and axial implants in either the prospective or retrospective studies subjected to review. The marginal bone loss observed with the tilted and axial implants likewise proved very similar. It thus can be deduced that tilted implants exhibit the same evolutive behavior as axial implants.

## References

[1] Block MS, Haggerty CJ, Fisher GR (2009). Nongrafting implant options for restoration of the edentulous maxilla. J Oral Maxillofac Surg.

[2] Krekmanov L, Kahn M, Rangert B, Lindström H (2000). Tilting of posterior mandibular and maxillary implants for improved prosthesis support. Int J Oral Maxillofac Implants.

[3] Aparicio C, Perales P, Rangert B (2001). Tilted implants as an alternative to maxillary sinus grafting: a clinical, radiologic, and periotest study. Clin Implant Dent Relat Res.

[4] Calandriello R, Tomatis M (2005). Simplified treatment of the atrophic posterior maxilla via immediate/early function and tilted implants: A prospective 1-year clinical study. Clin Implant Dent Relat Res.

[5] Maló P, Rangert B, Nobre M (2005). All-on-4 immediate-function concept with Brånemark System implants for completely edentulous maxillae: a 1-year retrospective clinical study. Clin Implant Dent Relat Res.

[6] Capelli M, Zuffetti F, Del Fabbro M, Testori T (2007). Immediate rehabilitation of the completely edentulous jaw with fixed prostheses supported by either upright or tilted implants: a multicenter clinical study. Int J Oral Maxillofac Implants.

[7] Testori T, Del Fabbro M, Capelli M, Zuffetti F, Francetti L, Weinstein RL (2008). Immediate occlusal loading and tilted implants for the rehabilitation of the atrophic edentulous maxilla: 1-year interim results of a multicenter prospective study. Clin Oral Implants Res.

[8] Agliardi EL, Francetti L, Romeo D, Del Fabbro M (2009). Immediate rehabilitation of the edentulous maxilla: preliminary results of a single-cohort prospective study. Int J Oral Maxillofac Implants.

[9] Balleri P, Ferrari M, Veltri M (2010). One-year outcome of implants strategically placed in the retrocanine bone triangle. Clin Implant Dent Relat Res.

[10] Agliardi E, Panigatti S, Clericò M, Villa C, Malò P (2010). Immediate rehabilitation of the edentulous jaws with full fixed prostheses supported by four implants: interim results of a single cohort prospective study. Clin Oral Implants Res.

[11] Hinze M, Thalmair T, Bolz W, Wachtel H (2010). Immediate loading of fixed provisional prostheses using four implants for the rehabilitation of the edentulous arch: a prospective clinical study. Int J Oral Maxillofac Implants.

[12] Francetti L, Romeo D, Corbella S, Taschieri S, Del Fabbro M (2012). Bone Level Changes Around Axial and Tilted Implants in Full-Arch Fixed Immediate Restorations. Interim Results of a Prospective Study. Clin Implant Dent Relat Res.

[13] Mattsson T, Köndell PA, Gynther GW, Fredholm U, Bolin A (1999 ). Implant treatment without bone grafting in severely resorbed edentulous maxillae. J Oral Maxillofac Surg.

[14] Rosén A, Gynther G (2007). Implant treatment without bone grafting in edentulous severely resorbed maxillas: a long-term follow-up study. J Oral Maxillofac Surg.

[15] Peñarrocha M, Carrillo C, Boronat A, Peñarrocha M (2010). Maximum use of the anterior maxillary buttress in severe maxillary atrophy with tilted, palatally positioned implants: a preliminary study. Int J Oral Maxillofac Implants.

[16] Celletti R, Pameijer CH, Bracchetti G, Donath K, Persichetti G, Visani I (1995). Histologic evaluation of osseointegrated implants restored in nonaxial functional occlusion with preangled abutments. Int J Periodontics Restorative Dent.

